# Thirty-fourth Session of the Georgia State Medical Association

**Published:** 1883-05-20

**Authors:** 


### THIRTY-FOURTH SESSION OF THE GEORGIA STATE MEDICAL ASSOCIATION.

### LETTER FROM ATHENS, GEORGIA.

*Messrs. Editors :*

The body convened in the court house at Athens, on Wednesday morning, April 18th. Dr. Holt, of Macon, in the chair. About fifty members present.

Dr. Gerdine, of Athens, delivered the address of welcome in his happiest style. Dr. Holt. the retiring president, introduced the present president, Dr. K. P. Moore, of Forsyth, who occupied the remainder of the morning session with a very interesting address on the subject of *Common Sense vs. Æstheticism.*

### AFTERNOON SESSION.

A few new members from Athens and the surrounding country were enrolled.

Resolutions of sympathy were passed for Dr. L. D. Ford, of Augusta, and Dr. J. T. Johnson, of Atlanta, in their long continued illness.

---

### SECOND DAY'S PROCEEDINGS.

The Association was called to order by the President, and the minutes read and approved.

The Secretary read letters from absent members.

The Secretary read an invitation from Mrs. Dr. C. W. Long, inviting the Association to call at her residence this evening from 7 to 8 o'clock.

On motion of Dr. Armstrong, the invitation was accepted, with the thanks of the Association.

On recommendation of the Board of Censors five new members were elected.

The committee on necrology made a report through Dr. Foster, their chairman. Report adopted.

The committee on prize essays made a report, which was adopted.

Voluntary contributions were called for, to be read by title, and to be called up at the pleasure of the Association.

A paper on Acute Dysentery, by Dr. J. W. Duncan, of Atlanta, was read.

Dr. Noble, of Atlanta, in behalf of Dr. Taliaferro, who was kept away by sickness, presented a number of instruments.

The report of the auditing committee was made through Dr. Holt, of Macon, and adopted.

A case was reported by Dr. Summey, of Stone Mountain, through Dr. Hamilton. The report was ordered sent to the committee on publications.

This was a case of "Pencil in the bladder." A pencil about five inches long was taken from the bladder of a young girl, around which was encrusted a phosphatic deposit, and the surmise by the reporter was that the PENCIL *had been swallowed nine months before the operation.* This ground was accepted as probable by about a half dozen members. The great majority, however, thought it more probable that the pencil entered through the urethra. It is fair to state that the girl had dysentery with bloody discharges shortly after it was claimed she had *swallowed* the pencil, but there was no evidence of any of the contents of the bowel having entered the bladder.

Dr. H. J. Williams read an article on typhoid fever.

A resolution by Dr. A. W. Griggs, of West Point, that the President of this Association appoint a committee of nine to memorialize the Georgia Legislature to amend the present laws in reference to dissection, to the end that our medical colleges may be placed on an equal footing with other colleges, was adopted.

The President appointed Drs. H. V. M. Miller, T. S. Powell, Wm. Perrin Nicolson, W. S. Armstrong, J. B. Baird, G. G. Crawford, W. F. Westmoreland, of Atlanta. Dr. DeSausure Ford, Augusta ; Dr. C. H. Hall, Macon.

A paper on typhoid fever, by Dr. L. G. Hardman, of Harmony Grove, was read.

The hour having arrived for the annual oration, Dr. A. W.

Griggs was appointed by the President to deliver the oration, in the absence of the orator elect.

The committee to nominate officers for the ensuing year was appointed.

Adjourned for dinner.

AFTERNOON SESSION.

The nominating committee reported the following officers for the ensuing year:

Dr. A. W. Calhoun, of Atlanta, President; Dr. R. J. Nunn, Savannah, first vice-president; Dr. M. P. Deadwyler, Elberton, second vice-president; Dr. Jas. A. Gray, Atlanta, secretary; Dr. A. W. Griggs, West Point, censor; Dr. J. S. Todd, Atlanta, censor; Eugene Foster, Augusta, censor.

A resolution was introduced by Dr. Holt, of Macon, returning thanks to the resident physicians of Athens for their kindness. To the faculty of the University for an invitation to visit the grounds, etc., of the University; to the principal of the Lucy Cobb, and also to Dr. Lipscomb.

The Association adjourned to meet in Macon on the 3d Wednesday in April, 1884.

The adjournment on the second day was unprecedented in the annals of the Association, with perhaps one exception. It was done late in the afternoon of the second day, when many were absent, and we think against the wish of the majority of the members.

A number of papers were ready and would have been read on the third day. It is to be hoped that the transactions will not be delayed ten or eleven months this year as they were last. All members, whether they attend the Association or not, have to pay three dollars annual dues, and it is certainly due them that they should receive this little book (the Transactions) earlier in the year than the eleventh month.

Altogether, this the 34th session of the Association might very well be blotted out of its records entirely, so far as its work as an Association is concerned, without much loss to the most of its members. But it is a dark cloud that has no clear spots. The social feature of the meeting was a grand success. The receptions and entertainments on Wednesday night, and the banquet on Thursday night, will ever be remembered with pleasure—thanks to the Doctors, and people generally, of the hospitable little city of Athens.

It is to be hoped that at the next meeting (in Macon) a chance will be afforded for more papers to be read.

"A MEMBER."

